# Early triggers of moderately high‐fat diet‐induced kidney damage

**DOI:** 10.14814/phy2.14937

**Published:** 2021-07-22

**Authors:** Andrea Sánchez‐Navarro, Miguel Ángel Martínez‐Rojas, Rebecca I. Caldiño‐Bohn, Rosalba Pérez‐Villalva, Elena Zambrano, Diana C. Castro‐Rodríguez, Norma A. Bobadilla

**Affiliations:** ^1^ Molecular Physiology Unit Instituto de Investigaciones Biomédicas Universidad Nacional Autónoma de México Mexico City Mexico; ^2^ Department of Nephrology Instituto Nacional de Ciencias Médicas y Nutrición Salvador Zubirán Mexico City Mexico; ^3^ Department of Biology of Reproduction Instituto Nacional de Ciencias Médicas y Nutrición Salvador Zubirán Mexico City Mexico; ^4^ CONACyT‐Cátedras Mexico City Mexico

**Keywords:** ER‐stress, glomerular hypertrophy, mitochondrial dynamics disruption, renal inflammation

## Abstract

Most of the obesity murine models inducing renal injury use calorie‐enriched foods, where fat represents 60% of the total caloric supply, however, this strategy doubles the standard proportion of fat ingestion in obese patients. Therefore, it is crucial to study the impact of a high‐fat intake on kidney physiology that resembles common obesity in humans to understand the trigger mechanisms of the long‐term consequences of overweight and obesity. In this study, we analyzed whether chronic feeding with a moderately high fat diet (MHFD) representing 45% of total calories, may induce kidney function and structural injury compared to C57BL/6 mice fed a control diet. After 14 weeks, MHFD induced significant mice obesity. At the functional level, obese mice showed signs of kidney injury characterized by increased albuminuria/creatinine ratio and higher excretion of urinary biomarkers of kidney damage. While, at the structural level, glomerular hypertrophy was observed. Although, we did not detect renal fibrosis, the obese mice exhibited a significant elevation of *Tgfb1* mRNA levels. Kidney damage caused by the exposure to MHFD was associated with greater oxidative stress, renal inflammation, higher endoplasmic reticulum (ER)‐stress, and disruption of mitochondrial dynamics. In summary, our data demonstrate that obesity induced by a milder fat content diet is enough to establish renal injury, where oxidative stress, inflammation, ER‐stress, and mitochondrial damage take relevance, pointing out the importance of opportune interventions to avoid the long‐term consequences associated with obesity and metabolic syndrome.

## Graphical abstract

Here, we demonstrated that obesity induced by a MHFD is enough to establish kidney damage, where oxidative stress, renal inflammation, ER‐stress, and mitochondrial damage take relevance, pointing out the importance of opportune interventions to avoid the long‐term consequences associated with obesity.

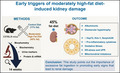

## 1 INTRODUCTION

The global overweight/obesity epidemic has worsened in the last decades, it has nearly tripled since 1975, affecting more than 1.9 billion adults and 350 million children in 2016, which has resulted in an increase in obesity‐related health complications with a significant impact on the economic burden of disease and mortality, accounting for 4 million deaths globally, most of them related to cardiovascular disease (Collaborators et al., 2017; Ogden et al., 2014; Tsuboi et al., 2017; Wang et al., 2008).

The higher cost of a balanced diet may explain why many families around the world face food insecurity and have a higher risk of developing overweight or obesity (Popkin et al., 2012). People choose less expensive foods that often have a high caloric density and low nutrient content, this has not only been reported in low‐ and middle‐income countries, but also in high‐income countries (FAO I, UNICEF, WFP, & WHO, 2020). It is well known that obesity predisposes to many cardiometabolic diseases, including type 2 diabetes mellitus (T2D), hypertension (HTN), chronic kidney disease (CKD), fatty liver disease, and several types of cancer, among others (Bluher, 2019; Eknoyan, 2011; Kovesdy et al., 2017; Tsuboi et al., 2017). In numerous large population‐based studies, having a higher body mass index (BMI) was associated with the reduction of estimated glomerular filtration rate (eGFR) (Chang et al., 2018; Foster et al., 2008; Pinto‐Sietsma et al., 2003; Tsuboi et al., 2017). In fact, when considering that the main causes of CKD are all related to high‐BMI states, we can highlight the impact of overweight and obesity in kidney health (Camara et al., 2017). The relationship between obesity and kidney damage is multifactorial and bidirectional, involving insulin resistance, hyperlipidemia, increased renin‐angiotensin‐aldosterone system activity, oxidative stress, chronic inflammation and mitochondrial dysfunction (Kovesdy et al., 2017; Takagi et al., 2018).

In most models of murine obesity in which kidney abnormalities have been reported, animals have free access to high‐calorie foods, where fat represents 60% of the total caloric intake, which is known as a high‐fat diet (HFD) and the follow‐up fluctuates between 12 and 34 weeks (Borgeson et al., 2017; Cheng et al., 2019; Kim et al., 2019; Luo et al., 2019; Pereira et al., 2020; Saito et al., 2019; Szeto et al., 2016; Xu et al., 2019; Yamamoto et al., 2017). Although this strategy leads effectively to the establishment of obesity in rodents and is capable of reproducing many of the multi‐organ alterations known in humans, it doubles the typical proportion of fat ingestion in obese patients (Botchlett & Wu, 2018). More realistic diets that resemble different degrees of obesity in humans are crucial to understand the trigger mechanisms of the long‐term consequences of overweight and obesity. Unfortunately, very little is known about calorie‐enriched foods containing a lesser content of fat on renal function in mice.

There is evidence that C57BL/6 mice fed with a diet containing 60% of fat for at least 12 weeks develop renal functional alterations like glomerular hyperfiltration and increased urinary excretion of albumin, as well as structural injury such as: glomerulopathy, podocytopathy and vacuolization of the tubular epithelium (Borgeson et al., 2017; Cheng et al., 2019; Kim et al., 2019; Luo et al., 2019; Pereira et al., 2020; Saito et al., 2019; Szeto et al., 2016; Xu et al., 2019; Yamamoto et al., 2017). These functional and structural abnormalities have been associated with increased reactive oxygen species (ROS) (Borgeson et al., 2017), endoplasmic reticulum stress (Li et al., 2019a), and more recently with disruption of mitochondrial homeostasis (Sun et al., 2020; Yamamoto et al., 2017). Since most of these studies have used the typical HFD, we decided to study the impact of chronic feeding of male C57BL/6 mice with a moderately high fat diet (MHFD) on kidney function and structure. We consider that this model resembles more accurately what happens to people with overweight, which is relevant in order to identify early pathophysiological events and find possible targets to prevent CKD development. We found that renal inflammation, oxidative stress, altered balance of mitochondrial fission/fusion, and endoplasmic reticulum stress (ER‐stress) are induced by feeding a MHFD in the mice for a relatively short period, showing deleterious outcomes of a moderate fat consumption in promoting renal injury.

## 2 METHODS

All experiments involving animals were conducted in accordance with the NIH Guide for the Care and Use of Laboratory Animals (https://grants.nih.gov/grants/olaw/guide‐for‐the‐care‐and‐use‐of‐laboratory‐animals.pdf) and with the Mexican Federal Regulation for animal reproduction, care, and experimentation (NOM‐062‐ZOO‐2001). The Animal Care Committee at Instituto Nacional de Ciencias Médicas y Nutrición Salvador Zubirán approved our experimental study.

### 2.1 Experimental protocol

Male C57BL/6 mice (Charles River Laboratories International, Inc.) aged 5–6 weeks and weighing 17–22 g were maintained in controlled conditions of temperature and humidity in our animal housing facility with 12:12 h day/night cycle, with free access to water and food. We did not use a method to generate the randomization sequence, the mice were only randomly assigned into two groups of at least 10 mice per group, as follows: (1) control mice fed with a control diet (C) and (2) obese mice (OB) fed with a moderately high fat diet (MHFD). The sample size was calculated according to the percentage of success and error test (Dell et al., 2002). The study was not blinded because no pharmacological intervention was carried out. All mice fill out our inclusion criteria and none was excluded. Both groups were follow‐up for 14 weeks. The experimental diets were homemade, as was previously reported by Castro‐Rodríguez et al. control diet contained 22.0% protein, 5.0% vegetable fat (corn oil), 31.0% polysaccharide, 31.0% simple sugars, 4.0% fiber, 6.0% minerals and 1.0% vitamins (w/w), and energy 4.0 kcal/g, in which fat represents 11.3% of caloric supply (Zeigler Rodent RQ 22–5). The MHFD contained 23.5% protein, 20.0% lard, 5.0% vegetable fat (corn oil), 20.2% polysaccharide, 20.2% simple sugars, 5.0% fiber, 5.0% minerals, 1.0% vitamins (w/w), and energy 4.9 kcal/g, where fat represents 45% of total caloric supply (Castro‐Rodriguez et al., 2020). Body weight was measured weekly and daily food consumption was recorded.

At the end of the experimental period, urine samples were collected over a 24‐h period in metabolic cages. Then, mice were anesthetized with sodium pentobarbital (30 mg/kg) to obtain blood samples by cardiac puncture. A slide of the kidney was fixed in paraformaldehyde 4% for histopathological analysis and both kidneys were weighed and preserved in liquid nitrogen (−80ºC) until analysis.

Urinary creatinine concentration was measured with the Quantichrom creatinine assay kit (DICT‐500).

### 2.2 Metabolic parameters and body composition

Analysis of body composition was performed in 6 animals per group using a 4‐ in‐1 small animal MRI (Echo Medical Systems). Serum analysis of glucose, triglycerides, and cholesterol were determined enzymatically with a SynchronCX auto analyzer (Beckman Coulter).

### 2.3 Hydrogen peroxide urinary excretion

The determination of urinary hydrogen peroxide as an oxidative stress marker was carried out, using a commercial kit (Amplex Red Hydrogen Peroxide/Peroxidase Assay, Roche, cat. no. A22188) following the manufacturer's instructions.

### 2.4 Albuminuria excretion

As a marker of kidney damage, urinary albumin concentration was analyzed by using a commercial kit Albuwell M (Exocell Inc., cat. no. 1011) following the manufacturer's instructions. This assay is a competitive ELISA completed in a direct mode, consisting of mouse‐specific albumin recognition by the provided horseradish peroxidase‐conjugated antibody, absorbance spectrophotometry, and concentration estimation using linear regression of a standard curve, and were normalized by urinary creatinine (UCr).

### 2.5 Detection of urinary biomarkers by Western Blot

Using Western blot technique, urinary HSP72 (Barrera‐Chimal et al., 2011; Morales‐Buenrostro et al., 2014; Ortega‐Trejo et al., 2015; Perez‐Villalva et al., 2017), KIM‐1 (Perez‐Rojas et al., 2007; Vaidya et al., 2005, 2006), and SerpinA3K (Sanchez‐Navarro et al., 2019) levels were detected in the diluted urine (1:10 in 0.9% saline solution) as follows: 10 μl of each dilution was loaded and resolved in 8.5% SDS‐PAGE. A mouse anti‐HSP72 (ENZO Life Sciences, cat. no. ADI‐SPA‐819F 1:5000 dilution), anti‐KIM‐1 (Boster, cat. no. PA1632, 1:5000) or anti‐SerpinA3K antibody (Proteintech, cat. no. 55480‐1‐AP, 1:1000) was incubated overnight at 4℃. Thereafter, membranes were incubated with a secondary antibody, HRP‐conjugated goat anti‐mouse IgG or anti‐rabbit IgG, respectively (Santa Cruz, cat. no. sc‐2031 and sc‐2004, respectively 1:5000). The proteins were detected using a commercial chemiluminescence kit (Millipore, Cat. No. WBKLS0500) and were normalized by urinary creatinine (UCr).

### 2.6 Western Blot and antibodies

The renal proteins were homogenized with a lysis buffer containing: 50 mM HEPES pH 7.4, 250 mM NaCl, 5 mM EDTA, 0.1% NP‐40 and complete protease inhibitor (Roche, cat. no. 11697498001). The proteins concentration was assessed by Lowry protein assay (Bio‐Rad, Cat. No. 5000113 and 5000114). Renal protein levels were detected by Western blot, tissue proteins (20 μg) were electrophoresed in a denaturing 8.5% acrylamide gel with SDS. The membranes were incubated with the primary antibody IL‐6 (Santa Cruz, cat. no. sc57315, 1:1000), BiP‐1 (Cell Signaling, cat. no. 3177, 1:1000), CHOP (Cell Signaling, cat. no. 2895, 1:5000), FOXO3 (Santa Cruz, cat. no. sc11351, 1:5000), Mitofusin‐1+Mitofusin‐2 (Abcam, cat. no. ab57602, 1:1000), Drp1 (Santa Cruz, cat. no. sc‐271583, 1:1000) and HRP β‐Actin antibody [AC‐15] (Abcam, cat. no. ab49900, 1:1,000,000) overnight at 4℃. Three of 10‐min washes were performed with TBS‐1x Tween, and then incubated with a secondary antibody coupled to HRP, anti‐rabbit or anti‐mouse IgG (Santa Cruz, cat. no. sc‐2031 or sc‐2004, respectively 1:5000). Tissue proteins assessed by Western blot were normalized by β‐actin detection.

#### 2.6.1 RNA extraction and quantitative PCR

Total RNA was isolated from whole kidneys using the TRIzol method (Invitrogen, cat. no. 15596026) and RNA integrity was evaluated using 1% agarose gel electrophoresis, to analyze rRNA. To avoid DNA contamination, total RNA samples were treated with DNase (DNase I; Invitrogen, cat. no. 18068015). Reverse transcription (RT) was carried out with 1 μg of total RNA and 200 U of Moloney murine leukemia virus reverse transcriptase (Invitrogen, cat. no. 18064022). The mRNA levels of Interleukin 6 (*Il*‐*6*), tumor necrosis factor alpha (*Tnfa*), interleukin 10 (*Il10*), transforming growth factor (*Tgfb1*) vascular endothelial growth factor (*Vegfa*), catalase (*Cat*), superoxide dismutase (*Sod2*) and glutathione peroxidase 1 (*Gpx1*) were quantified by real‐time PCR on an ABI Prism 7300 Sequence Detection System (TaqMan, ABI, Foster City, CA, cat. no. 4331182). Probes were ordered as follows: *Il*6 (Mm00446190_m1), *Tnfa* (Mm0443258_m1), *Il10* (Mm01288386_m1), *Tgfb1* (Mm03024053_m1), *Vegfa* (Rn01511602_m1), *Cat* (Mm00437992_m1), *Sod2* (Mm01313000_m1) and *Gpx1* (Mm00656767_g1). As an endogenous control, eukaryotic *18S* rRNA (predesigned assay reagent Applied by ABI, external run, Rn03928990_g1, Cat. No. 4319413E) was used. The relative quantification of each gene expression was performed using the comparative threshold cycle (Ct) method.

#### 2.6.2 Histopathological analysis

After tissue fixation, the kidneys were dehydrated and embedded in paraffin. Renal slices of 4 μm were obtained and stained with Periodic Acid Schiff (PAS). Twelve high‐power fields (Magnification 200×) were captured from the renal cortex of each kidney to ensure the presence of at least 12 glomeruli per kidney using a camera incorporated onto the microscope. The glomerulus size was evaluated by measuring glomerular area in each captured glomerulus per mouse using the NIS‐Elements software (Nikon Instruments Inc.). Because glomeruli are spheroidal in shape and in each section of the kidney, each glomerulus can be cut at the lower, upper, or middle pole, the glomerular area was organized by ranks and then the differences between the C and MHDF groups were determined by a contingency analysis, as we have previously reported (Barrera‐Chimal et al., 2013, 2018; Garcia‐Ortuno et al., 2019; Perez‐Rojas et al., 2007). Researchers were blind to the experimental group.

#### 2.6.3 Statistical analysis

The results are presented as the mean ± SE. The significance of the differences between groups was assessed by unpaired *t*‐test. All comparisons passed the normality test. The differences in the ranks of glomerular area among the groups were evaluated by contingency analysis, and the differences were assessed using the chi‐squared test with Yates correction. Statistical significance was defined when the *p* value was <0.05. All the graphs and statistical analyses were performed using the statistical GraphPad Prisma 8 software for Mac (GraphPad Software).

## 3 RESULTS

### 3.1 Overweight and renal damage induced by a MHFD

**FIGURE 1:**
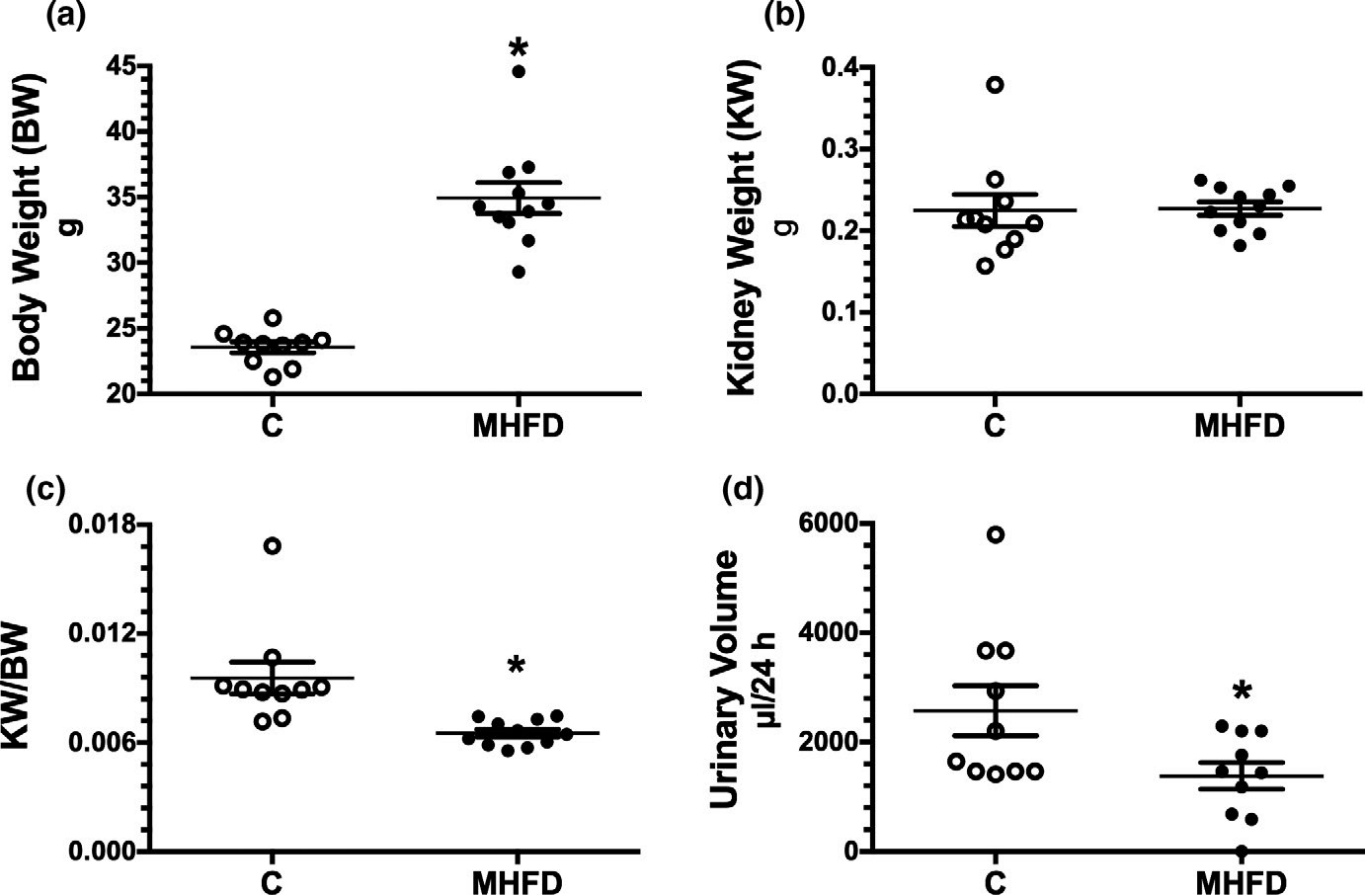
Physiological parameters induced by a MHFD at the end of the study. (a) Body weight (BW) and (b) kidney weight (KW) (g), respectively. (c) Kidney weight/body weight ratio (KW/BW). (d) urinary volume (µl/24 h). The group fed with a control diet (c) is represented in white circles, while the MHFD group in black circles, *n* = 10/11 per group, respectively for (a), (b), (c) and *n* = 10 per group for (d). Lines stated in each group represent the mean ± SE. *p < 0.05 versus the C group assessed by unpaired *t*‐test

The experimental group was fed with a MHFD for fourteen weeks. We observed that the MHFD group exhibited an increase in body weight (BW) by 48% compared to the control group (Figure 1a), without changes in the kidney weight (KW) (Figure 1b). In addition, no statistical difference was observed in the ratio kidney weight g/lean % (0.0034 ± 0.0002 vs. 0.0026 ± 0.0003, respectively, *p* = NS). However, the ratio kidney/body weight (KW/BW) was reduced (Figure 1c). We also observed that the MHFD group had decreased urine output (Figure 1d).

In Table S1 (https://figshare.com/s/753eb0b268daba362bf6) (https://doi.org/10.6084/m9.figshare.14224199) appears the corporal composition and metabolic parameters for the studied groups. Serum glucose and cholesterol levels were significantly increased by 35.6% and 45.1%, respectively in the MHDF group. In the MHFD group there was an increase in fat tissue percentage compared to the control group (33.6% vs. 8.4%, respectively). Similarly, the visceral adipose tissue was greater in the MHFD than in the C group (2.9 vs. 0.4 g, respectively). These alterations resulted in a reduction in lean tissue percentage in the MHDF compared to the control group (66.2% vs. 91.2%, respectively). At the end of the study, the MHFD group exhibited mild metabolic dysfunction characterized by a significant increase in serum glucose and in cholesterol, without changes in triglycerides levels.

**FIGURE 2:**
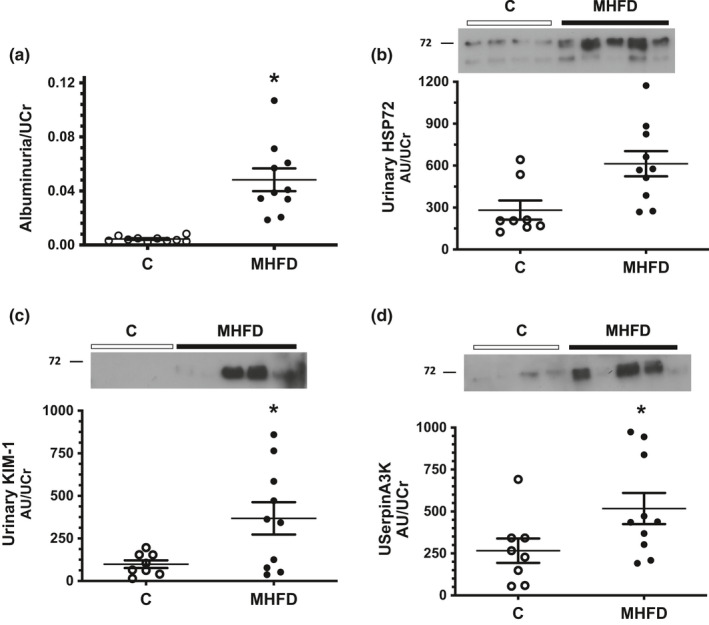
Kidney biomarkers of renal injury. (a) Albuminuria/UCr evaluated by ELISA (µg/ml). (b, c, and d) Urinary HSP72, urinary KIM‐1, and urinary SerpinA3K respectively, detected by WB analysis and corrected by urinary creatinine (AU/UCr), in these graphs insets of representative blots are included. The group fed a control diet (C) is represented in white circles, while the MHFD group in black circles, *n* = 8/10 per group, respectively for (b), (c), (d) and *n* = 10 per group for (a). Lines reflect mean ± SE. *p < 0.05 versus the C group assessed by unpaired *t*‐test

Renal dysfunction induced by MHFD was evaluated by albuminuria/UCr (Figure 2a) and urinary biomarkers of kidney injury: HSP72 (Figure 2b), KIM‐1 (Figure 2c), and serpinA3K (Figure 2d). The HFD group exhibited a significant increase in albuminuria/UCr compared to the C group (0.048 vs. 0.004, respectively). In accordance with these results a significant increase in UHSP72, UKIM‐1 and UserpinA3K was observed in the MHFD group by 2.1, 3.7 and 1.9‐fold, respectively, compared to the control group.

**FIGURE 3:**
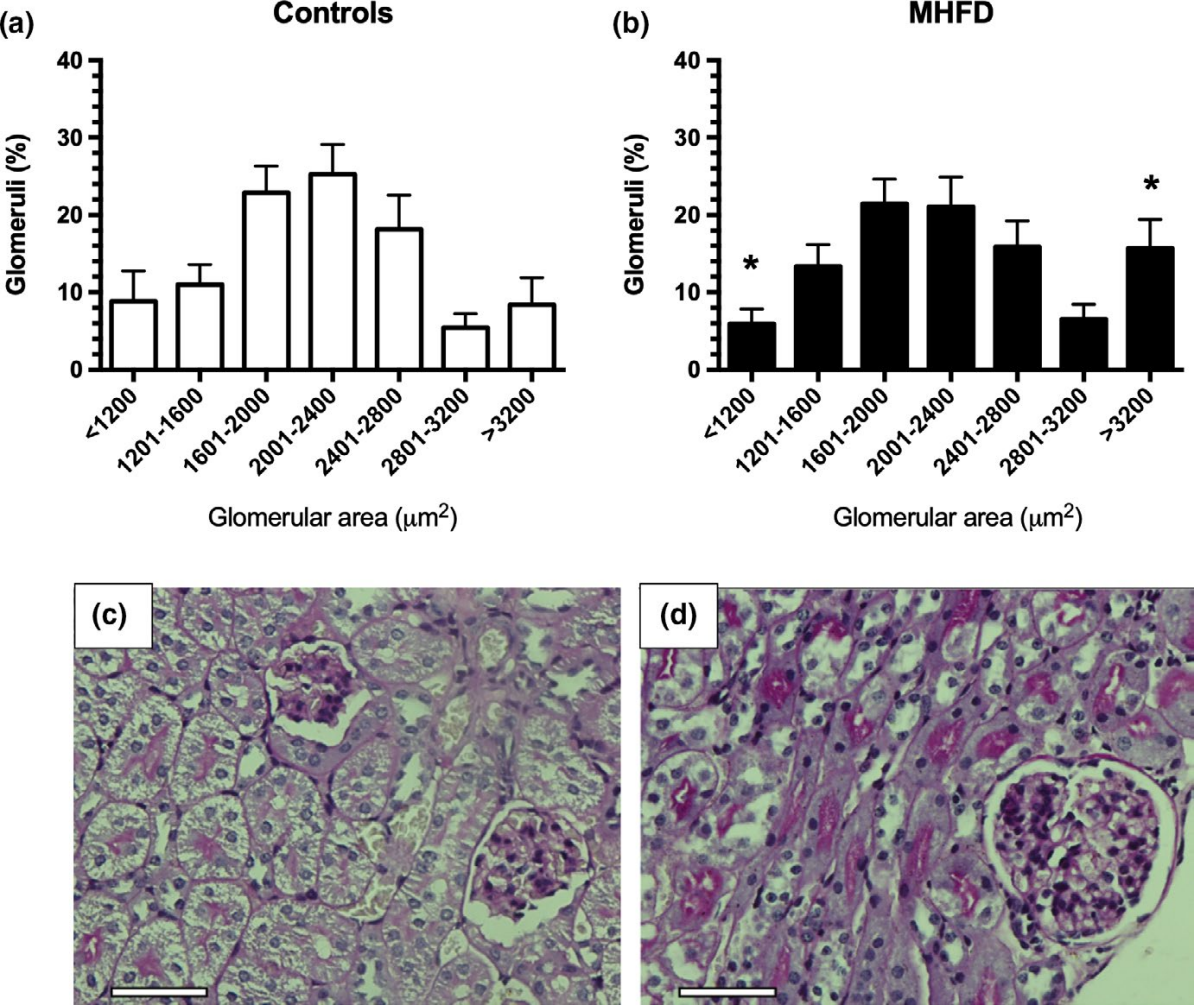
Glomerular hypertrophy induced by MHFD. (a,b) Glomerular area distribution along seven ranks are represented in white bars for the control diet group (a), and in black for MHFD group (b). (c,d) Representative micrographs staining with PAS showing glomerular hypertrophy induced by MHFD. Scale bar 50 μm (Original magnification 200×). *p < 0.01 versus the C group assessed by a contingency analysis

Because hyperfiltration and glomerular injury are associated with albuminuria in obese subjects (D'Agati et al., 2016), we quantified glomerular area in at least 12 glomeruli of each mouse. In seven ranges, the glomerular areas were distributed, the percentage in each range was calculated and the statistical differences between the C and MHDF groups were analyzed by contingency analysis (Figure 3). Interestingly we found a higher proportion of large glomeruli (>3200 μm^2^) in the kidney microphotographs from obese mice, whereas the C group exhibited a normal distribution in the glomerular areas, where most of the glomeruli were between 2001 and 2400 μm^2^ (Figure 3b), indicating that glomerular hypertrophy is part of the renal structural abnormalities in our model. To address the establishment of chronic kidney disease, we also evaluated fibrotic area using Sirius Red stain, as a final common pathway of chronic kidney injury; however, we did not find extracellular matrix (ECM) expansion in any of the obese mice compared to the C group (Figure 3e,f). These results indicated that mice fed with a MHFD exhibited clear signs of early renal injury.

### 3.2 Mice fed a MHFD exhibited renal inflammation, oxidative stress and ER‐stress

**FIGURE 4:**
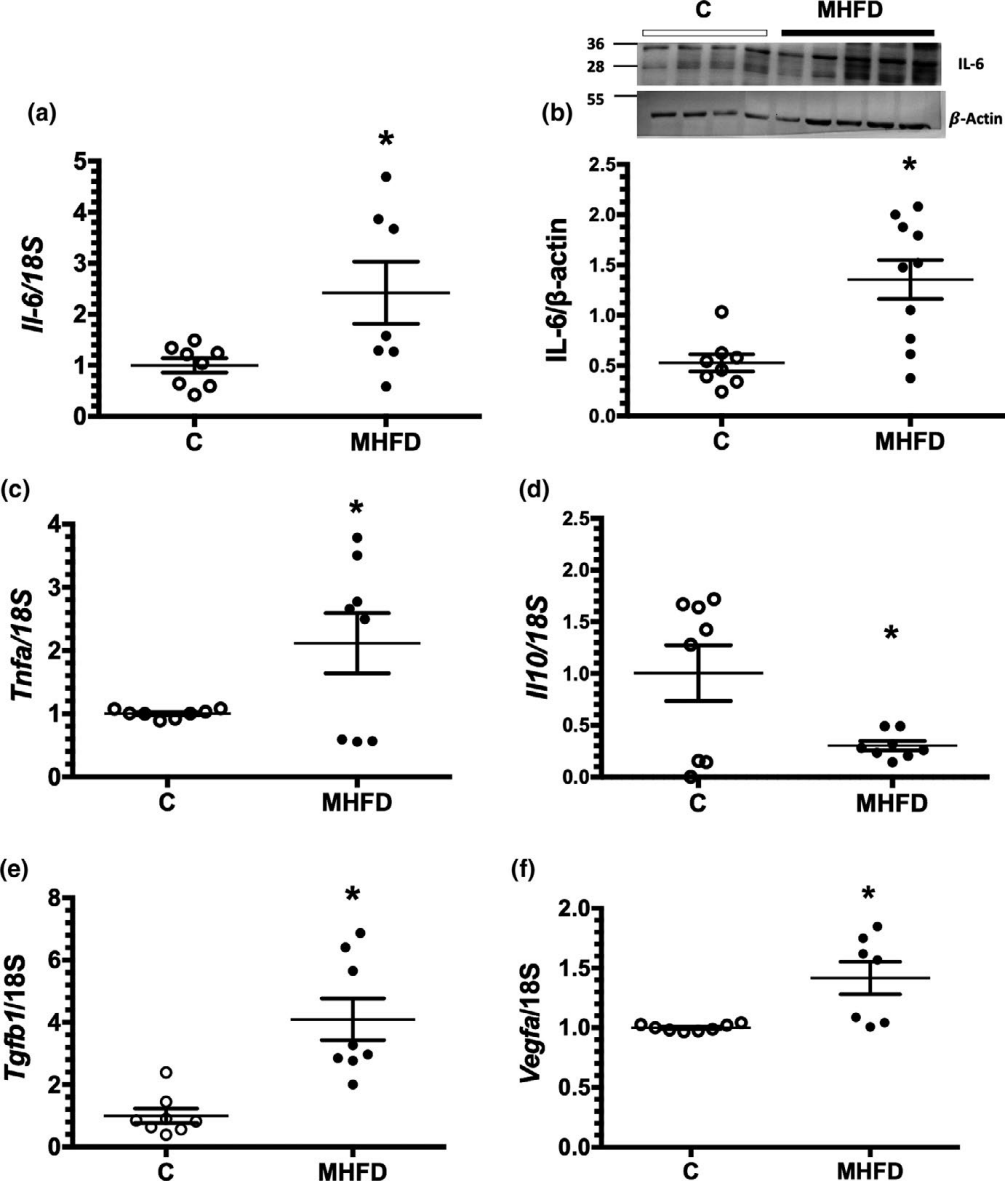
Kidney inflammation induced by MHFD. (a) Relative expression of *Il*‐*6* mRNA and (b) Protein levels of IL‐6, including a representative blot. (c) Relative expression of *Tnfa* mRNA levels. (d) Relative expression of *Il*‐*10* mRNA levels. (e) Relative expression of *Tgfb1* mRNA levels and (f) Relative expression of *Vegfa* mRNA levels. In the IL‐6 detection by WB: the first four samples corresponded to the control group (C) and the remaining five samples are from the MHFD mice and corrected by β‐actin. The group fed a control diet (C) is represented in white circles, while the MHFD group in black circles, *n* = 8/10 per group, respectively for (b) and *n* = 8 per group for (a), (c), (d), (e), (f). Lines reflect mean ± SE. *p < 0.05 versus the C group assessed by unpaired *t*‐test

Obese mice fed a MHFD exhibited inflammation as is shown by the increase in interleukin‐6 (*Il6*) mRNA (Figure 4a) and protein levels (Figure 4b), as well as by the increase in *Tnfa* mRNA levels (Figure 4c), and a significant reduction were observed in the anti‐inflammatory cytokine: interleukin‐10 (*Il10*) (Figure 4d). Interestingly, transforming growth factor β (*Tgfb1*) mRNA levels were significantly increased in the kidney of obese mice (Figure 4e). In addition, significant increase of vascular endothelial growth factor (*Vegf*) mRNA levels was observed in the HFD group compared to the C group (Figure 4f).

**FIGURE 5:**
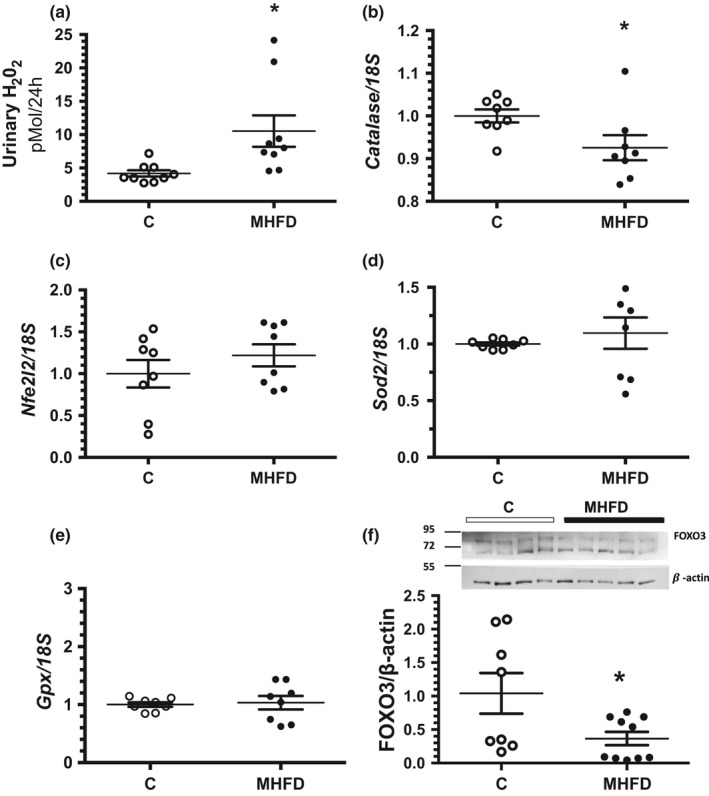
Oxidative stress after 14 weeks of MHFD. (a) Urinary Hydrogen Peroxide (Urinary H_2_O_2_) (b–e) Relative expression of *Catalase*, *Nrfe2l2*, *Sod2*, and *Gpx*, respectively (f) Protein levels of FOXO3 and β‐actin detected by WB including the insets of a representative blot. The first four samples correspond to the control group (C) and the remaining five to the MHFD group, the graph represents FOXO3 corrected by β‐actin. The group fed a control diet (C) is represented in white circles, while the MHFD group in black circles, *n* = 9 per group for (a); *n* = 8 per group for (b), (c), (d), (e) and *n* = 8/10 per group, respectively for (f). Lines reflect mean ± SE. *p < 0.05 versus the C group assessed by unpaired *t*‐test

Oxidative stress was indirectly evaluated by urinary hydrogen peroxide levels (Urinary H_2_O_2_) and by antioxidant enzymes mRNA levels. Urinary H_2_O_2_ was clearly enhanced in the MHFD group compared to the C group (10.5 ± 2.3 vs. 4.2 ± 0.5 pMol/24 h) (Figure 5a). Although, we did not observe changes in nuclear factor erythroid 2 Like 2 (*Nfe2l2*) mRNA levels between the studied groups (Figure 5b), catalase (*Cat*) mRNA levels were significantly reduced in the MHFD group compared to the C group (Figure 5c), whereas no changes in superoxide dismutase 2 (*Sod2*) or glutathione peroxidase (*Gpx*) mRNA levels were found (Figure 5d,e). We also evaluated the protein levels of FOXO3, because of its critical role in the regulation of metabolism, oxidative stress and hypoxic response, as well as, its role in the cell‐cycle (Senf et al., 2011). We found a significant reduction in FOXO3 protein levels in the obese animals, consistent with the oxidative stress found in the MHFD group (Figure 5f).

**FIGURE 6:**
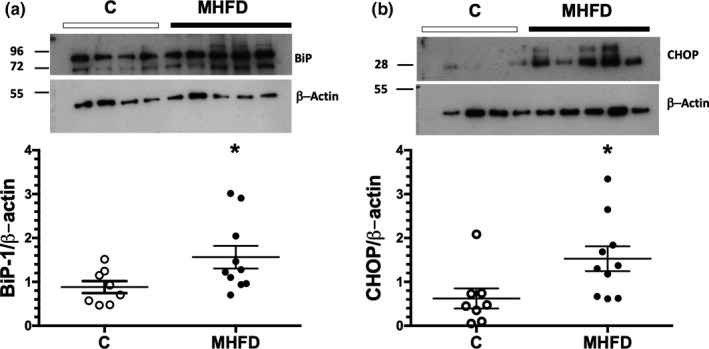
Renal endoplasmic reticulum stress induced by a MHFD in the mice. (a) Protein levels of BIP‐1 and (b) Protein levels of CHOP, including representative blots. In the WB image inset: the first four samples correspond to the controls (C) and the remaining five to the MHFD mice group and corrected by β‐actin. The bars reflect mean ± SE. *p < 0.05 versus control group. The group fed a control diet (C) is represented in white circles, while the MHFD group in black circles, *n* = 8/10 per group, respectively. Lines reflect mean ± SE. *p < 0.05 versus the C group assessed by unpaired *t*‐test

Additionally, we studied the protein levels of BiP‐1 and CHOP involved in ER‐stress in response to energetic or cellular stress. A significant increase by almost 2‐fold in BiP‐1 was observed in the MHFD group compared to the C group (Figure 6a). Similarly, a higher increase by 2.5‐fold was found in CHOP expression in the MHFD group compared to the C group (Figure 6b).

### 3.3 Obese mice exhibited disruption of mitochondrial dynamics

**FIGURE 7:**
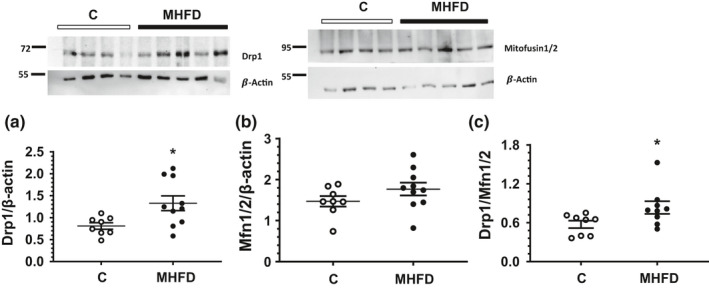
Altered balance of mitochondrial fission/fusion in the kidney of MHFD mice. (a) Protein levels of Drp1 and (b) Protein levels of Mitofusin1, including a representative blot. (c) Ratio Drp1/Mitofusin1. In the WB image insets: the first four samples correspond to the controls (C) and the remaining five are from the MHFD mice group and corrected by β‐actin. The bars reflect mean ± SE. *p < 0.05 versus control group. The group fed a control diet (C) is represented in white circles, while the MHFD group in black circles, *n* = 8/10 per group, respectively. Lines reflect mean ± SE. *p < 0.05 versus the C group assessed by unpaired *t*‐test

Finally, we evaluated the mitochondrial dynamics by analyzing the protein levels of Mitofusin‐1+Mitofusin‐2 (Mfn1/2) and Dynamin‐related Protein‐1 (Drp1) by western blot, as subrogates of mitochondrial damage. We found a significant increase in Drp1 protein levels (Figure 7a), whereas Mfn1/2, a basic component of the mitochondrial outer membrane fusion machinery, did not change (Figure 7b). Nevertheless, we found an increased Drp1‐to‐Mfn1/2 expression ratio in the animals fed a MHFD (Figure 7c). These results reflect the increased mitochondrial fragmentation and damage, that could be related to the oxidative and ER‐stress.

## 4 DISCUSSION

In this study, we demonstrated that feeding male mice with a lesser content of fat (45%) for a relatively short period induced relevant renal alterations characterized by a significant increase in albuminuria, glomerular hypertrophy, and increased excretion of urinary kidney injury biomarkers (Hsp72, Kim1, and serpinA3), in spite of a discretional metabolic impairment. All these abnormalities were associated with altered balance of mitochondrial fission/fusion, renal inflammation, and increased oxidative and ER‐stress.

Despite the efforts that have been made to study the mechanisms involved in the relationship between obesity and renal damage, there is an inadequate understanding of the crucial events that lead to long‐term alterations in the kidney. A high‐fat diet induces an initial adaptation of mitochondrial bioenergetics to balance the energy supply and demand, in order to optimize nutrient overload and consumption. However, this adaptation is accompanied by a reduced respiratory efficiency, increased proton leakage, ROS generation and increased mito‐autophagy (Garrison et al., 2017; Ruggiero et al., 2011). Accordingly, we found that mice fed a MHFD exhibit greater oxidative stress that was evidenced by a significant increase in urinary H_2_O_2_ excretion and a significant reduction in catalase mRNA levels. In addition, FOXO3 protein levels were reduced in the kidney from the MHFD group, which could contribute to more ROS generation. In this regard, it has been shown that the conditional deletion of FOXO3 exacerbates acute kidney injury (AKI) to CKD transition by reducing epithelial autophagy and lowering SOD2 expression. On the contrary, FOXO3 activation in hypoxic tubules prevents CKD (Li et al., 2019b).

Although we did not measure GFR, the presence of glomerular hypertrophy suggests an increase in intraglomerular pressure (D'Agati et al., 2016), which leads to enhanced solute and fluid delivery along the nephrons, therefore increasing metabolic demand, and possibly contributing to oxidative stress perpetuation. The presence of albuminuria and the increased excretion of tubular injury biomarkers in the MHDF group suggest a deterioration of both, glomerular filtration barrier and tubular epithelium.

Factors associated with obesity, like elevated circulating levels of free fatty acids (FFA) can lead to ER‐stress in several tissues. ER is essential for the biosynthesis of proteins, lipid metabolism and regulation of calcium metabolism (Martin‐Jimenez et al., 2017). However, the precise mechanisms that lead to ER‐stress induced by obesity, is still unknown. Recent evidence suggests that ER‐stress is dependent on dysregulation of calcium homeostasis, increased ROS, and FFA accumulation (Cnop et al., 2012; Martin‐Jimenez et al., 2017). In HFD‐fed mice, excessive ectopic accumulation of FFA has been demonstrated in several tissues, leading to cellular lipotoxicity, remarkable ER‐stress, and an activated unfolded protein response (UPR) (Li et al., 2019a; Tanaka et al., 2012). Indeed, UPR becomes activated with the accumulation of misfolded proteins in the ER lumen (Martin‐Jimenez et al., 2017) that intends to balance the ER functional capacity. Additionally, oxidative and ER‐stress are closely interrelated phenomena because oxidative stress can disturb the ER redox balance leading to the disruption of disulfide bonds, the misfolding of proteins and the generation of ROS (Cao & Kaufman, 2014; Victor et al., 2021).

Moreover, it has been shown that ER‐stress contributes to the development of insulin resistance and inflammation. In fact, ER‐stress preceded inflammation in the liver mediated by lipotoxicity (Martin‐Jimenez et al., 2017; Ozcan et al., 2004; Townsend et al., 2019). During ER‐stress, protein kinase RNA‐like ER kinase (PERK), inositol‐requiring protein 1α (IRE1α) and activating transcription factor 6 (ATF6) dissociate from the ER chaperone immunoglobulin heavy chain‐binding protein (BiP), which then increases its binding to misfolded proteins found in the lumen (Cnop et al., 2012; Martin‐Jimenez et al., 2017). Consistent with this, we found a significant increase in BiP expression, suggesting that feeding the mice a MHFD for 14 weeks is enough to induce renal ER‐stress. The BiP up‐regulation is associated with the prevention of protein translation, and inefficient removal of misfolded proteins, which favors apoptosis of damaged cells, as an adaptive mechanism (Dandekar et al., 2015; Kim et al., 2018a). Furthermore, in chronic ER‐stress, signaling switches from pro‐survival to pro‐apoptosis by upregulating the C/EBP homologous protein (CHOP) driven by ATF4 associated to the PERK/eIF2‐α pathway to trigger apoptosis (Groenendyk et al., 2010; Li et al., 2019a). Chronic ER‐stress also leads to oxidative stress and inflammation (Kim et al., 2018b). In fact, in human obesity and models of genetic or dietary obesity, ER‐stress leads to the activation of UPR and the expression of CHOP. The upregulation of CHOP has been related with the induction of inflammation, leading to lower number of macrophages‐M2 and lymphocytes Th2, well known to establish and anti‐inflammatory response (Suzuki et al., 2017). In human obesity, UPR pathways' activation, evaluated by CHOP and BiP upregulation, happens together with oxidative stress and inflammation (Banuls et al., 2017; Komura et al., 2010; Sage et al., 2012) this data supports the existence of cross‐linked mechanisms that occur in the kidneys as a result of obesity.

There is also evidence that ER‐stress also leads to inflammation through the liberation of Ca^2+^ and ROS from the ER that trigger the Toll‐like receptor (TLR) pathways, including nuclear factor κB (NF‐κB), mitogen‐activated protein kinase (MAPK), and glycogen synthase kinase‐3β (GSK‐3β) (Victor et al., 2021). Accordingly, we found that the MHFD group exhibited an increase in interleukin‐6 mRNA and protein levels, as well as an increase in *Tnfa* mRNA levels. Additionally, *Tgfb1* mRNA levels were significantly increased too. This cytokine is well known to be associated with renal fibrosis by promoting the proliferation and the activation of interstitial fibroblasts and the epithelial‐mesenchymal transition (Iwano et al., 2002; Lovisa et al., 2015). Although, we did not find ECM expansion, the elevation of *Tgfb1* could represent the initiation of a pro‐fibrotic state into the kidney, which could be histologically evidenced in future studies with a longer follow‐up.

In this study, we also found a significant increase in *Vegf* mRNA levels in the MHFD group. VEGF‐A is a protein that can be secreted by podocytes and its function is critical for podocytes, mesangial and endothelial cells survival. VEGF‐A regulates slit‐diaphragm signaling and podocyte shape through VEGF receptor 2‐nephrin‐actin interactions. Previous studies have demonstrated that chronic hyperglycemia induced an excess of podocyte VEGF‐A expression and low endothelial nitric oxide (NO) generation. The abnormal crosstalk between VEGF‐A and NO pathways results in an increased oxidative stress (Tufro & Veron, 2012). Moreover, elevation of VEGF alone reproduces some aspects of glomerulopathy, and its antagonism attenuates diabetic albuminuria and other associated features of the podocytopathy (Cooper et al., 1999; Sung et al., 2006).

Emerging evidence suggest that dysfunctional mitochondria have a primary role in the development of CKD. There are some hallmark features of mitochondrial dysfunction, such as, changes in morphology, remodeling, and decrease in both mitochondrial biogenesis and ATP production (Galvan et al., 2017). Considering that we found an interesting bioenergetic dysregulation, reflected by the oxidative and ER‐stress, we decided to analyze the mitochondrial dynamics as subrogate of mitochondrial dysfunction. Under normal conditions, both mitochondrial fission and fusion occur continuously and are precisely controlled by associated modulators (Chan, 2012). We found that Drp1 protein levels, that is a member of the GTPase family, and controls the final step in mitochondrial fission (Correa‐Rotter & Gamba, 1997), was significantly elevated in the MHFD group, while Mfn1/2, a basic component of the mitochondrial fusion machinery did not change (Chan, 2012). Therefore, our results suggest that increased mitochondrial fragmentation is another implicated mechanism in renal injury induced by a MHFD (Jeong et al., 2018). In this regard, increased numbers of fragmented mitochondria have been observed in renal diseases, such as diabetic nephropathy (Brooks et al., 2009; Lee et al., 2017).

In summary, this model of MHFD‐induced kidney damage has given us a great opportunity to detect key initiators, suggesting relevant interactions that can lead to the development of CKD. At the same time, it has taught us the possible kidney disorders that can occur in obese patients. We also believe that this model could be a useful tool to study the role of antioxidant molecules and drugs to protect ER and mitochondrial dynamics as new therapeutic targets for obesity‐induced kidney damage.

## CONFLICT OF INTEREST

The authors declare no conflict of interest.

## AUTHOR CONTRIBUTIONS

Conception and design: EZ and NAB. Inclusion and following of experimental mice, and metabolic analysis: DCCR and EZ. Performed renal functional and structural analysis, as well as biochemical and molecular assessment: ASN, MAMR, RICB, and RPV. Acquisition of data: ASN, and MAMR. Analysis and interpretation of data: ASN, MAMR, and NAB. Drafting the article and revising it critically for important intellectual content: N.A.B, A.S.N. and MAMR.

## Supporting information



Supplementary Material

Table S1
