# Supplementary material for: Early triggers of moderately high‐fat diet‐induced kidney damage
Source: Physiol Rep. 2021 Jul 22;9(14):e14937. doi: 10.14814/phy2.14937 (PMC8295594; doi:10.14814/phy2.14937)
Supplement: Supplementary file 1 — Supplementary Material [file PHY2-9-e14937-s001.pdf]

Urinary HSP72
MW=72KDa

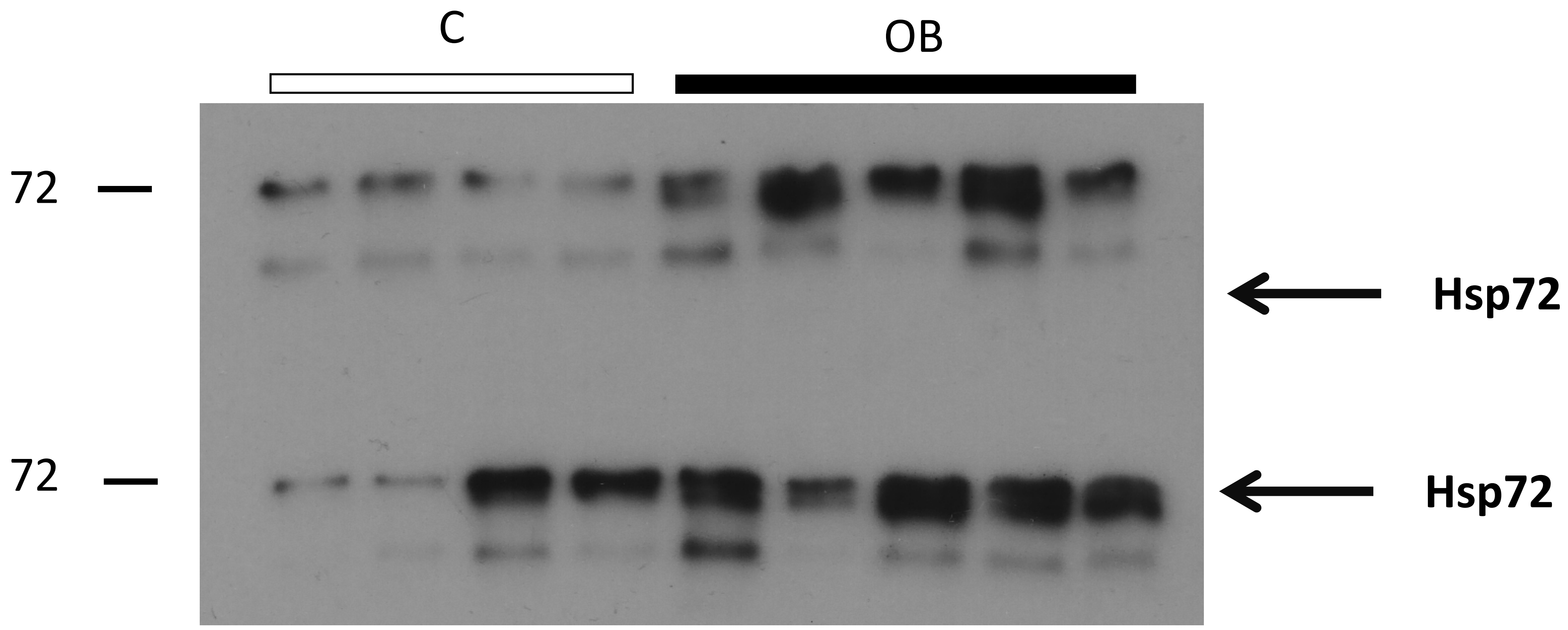

HSP72
10ul urine
1:5,000
1:5,000 ms

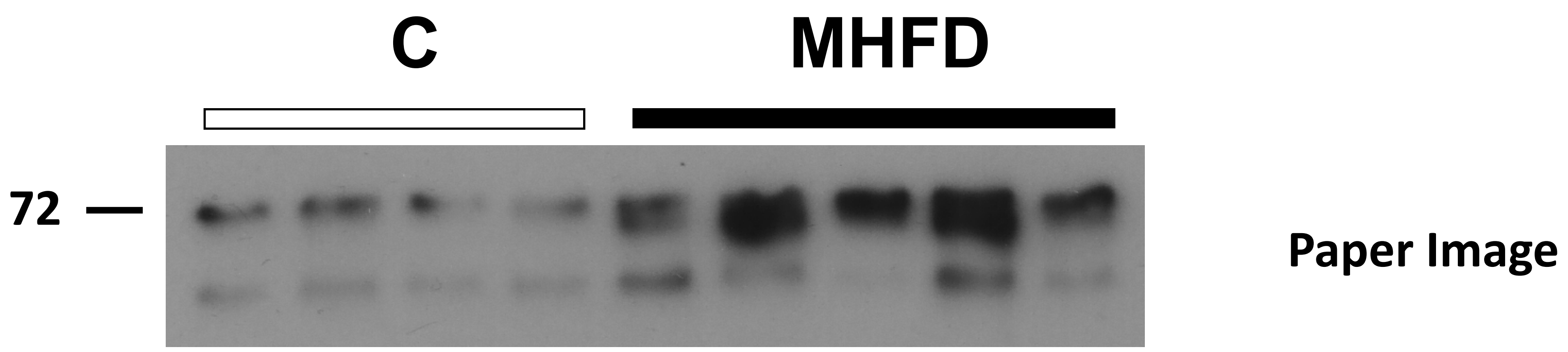

Urinary KIM-1
MW=55KDa

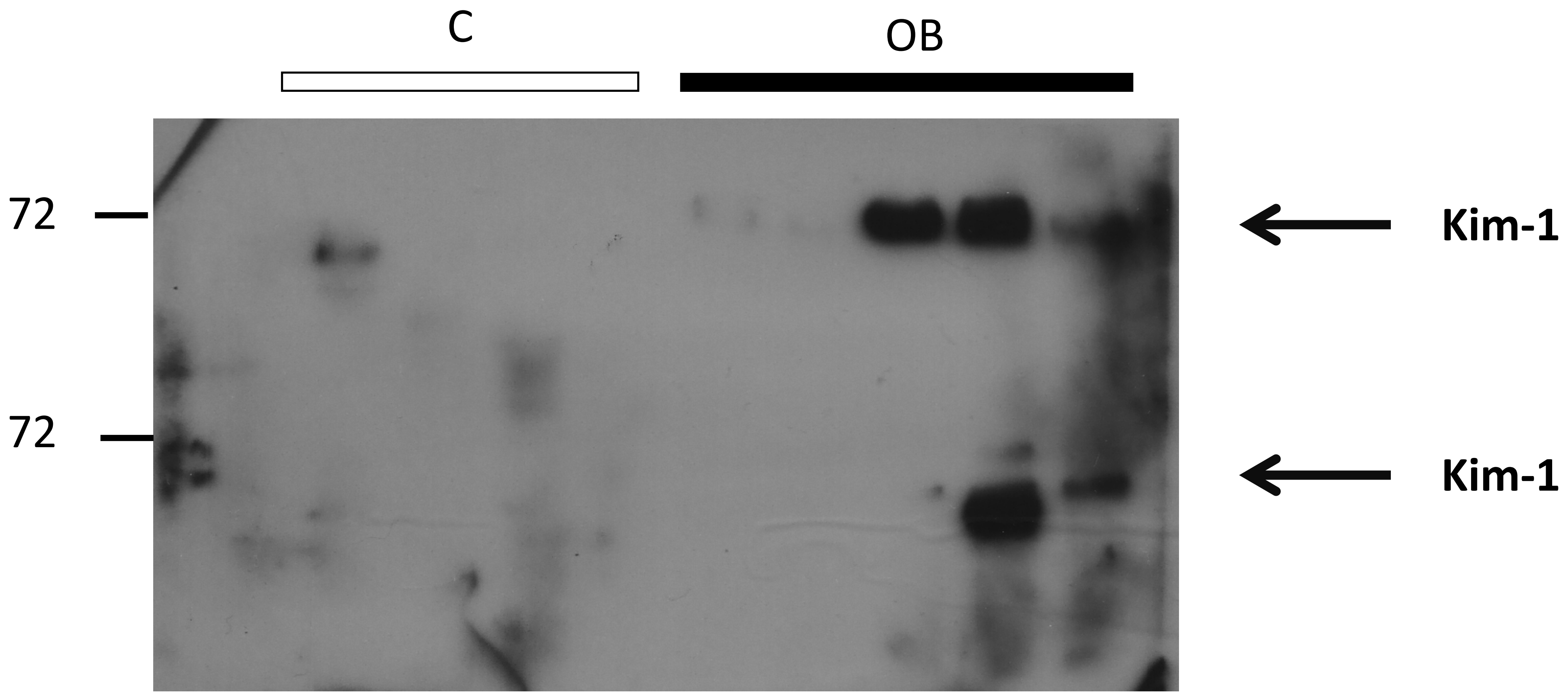

KIM-1
10ul urine
1:5,000
1:5,000 rb

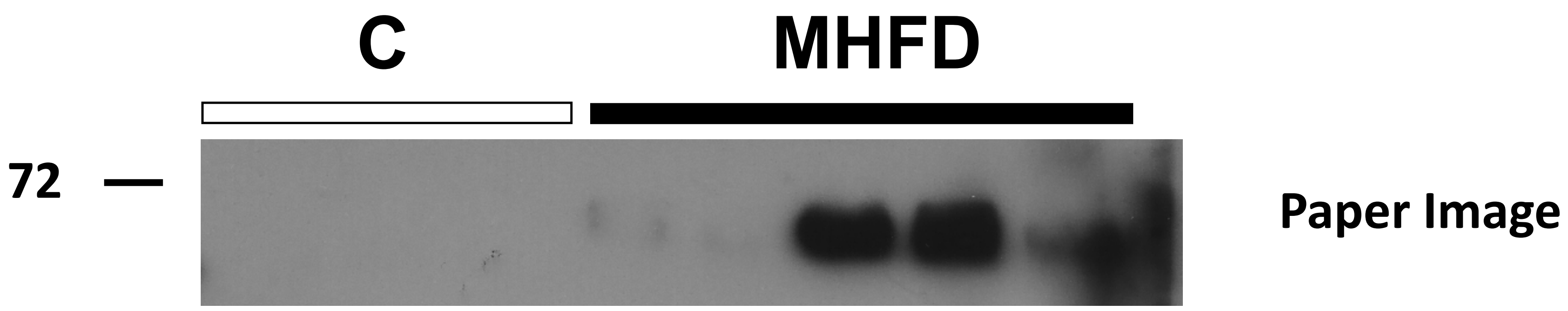

Urinary SerpinA3K
MW=55-72KDa

SerpinA3K
10ul urine
1:1,000
1:5,000 rb

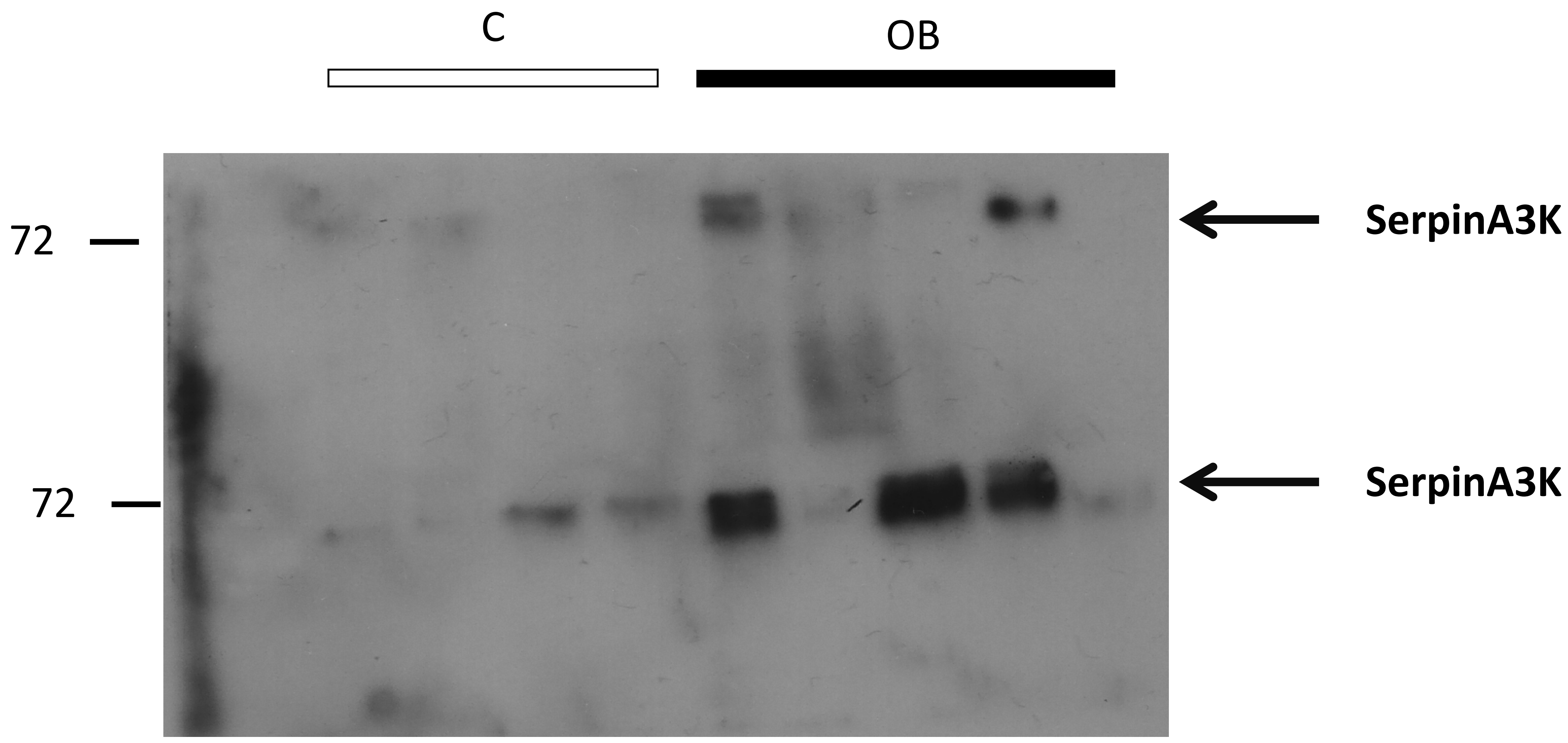

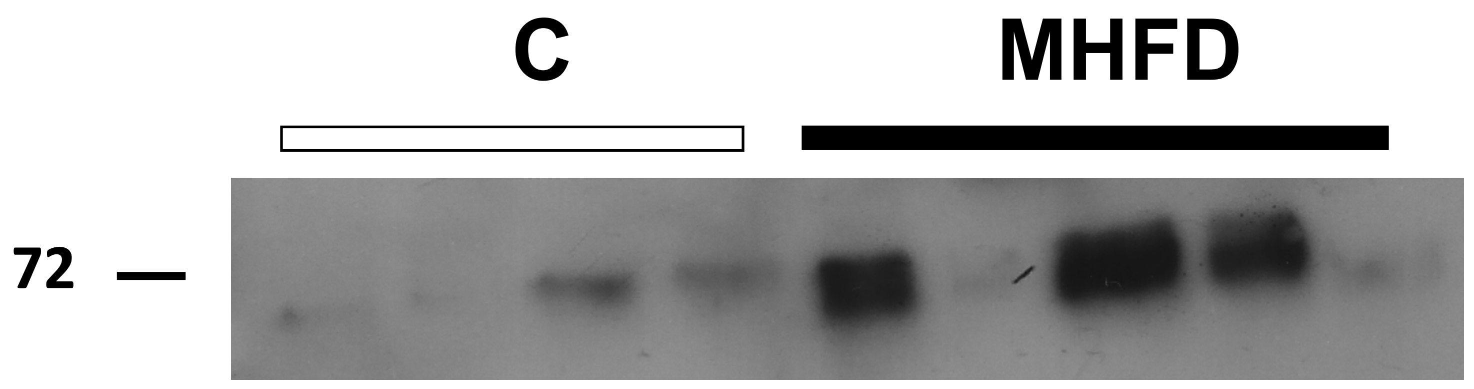

Paper Image

BiP
MW=78KDa

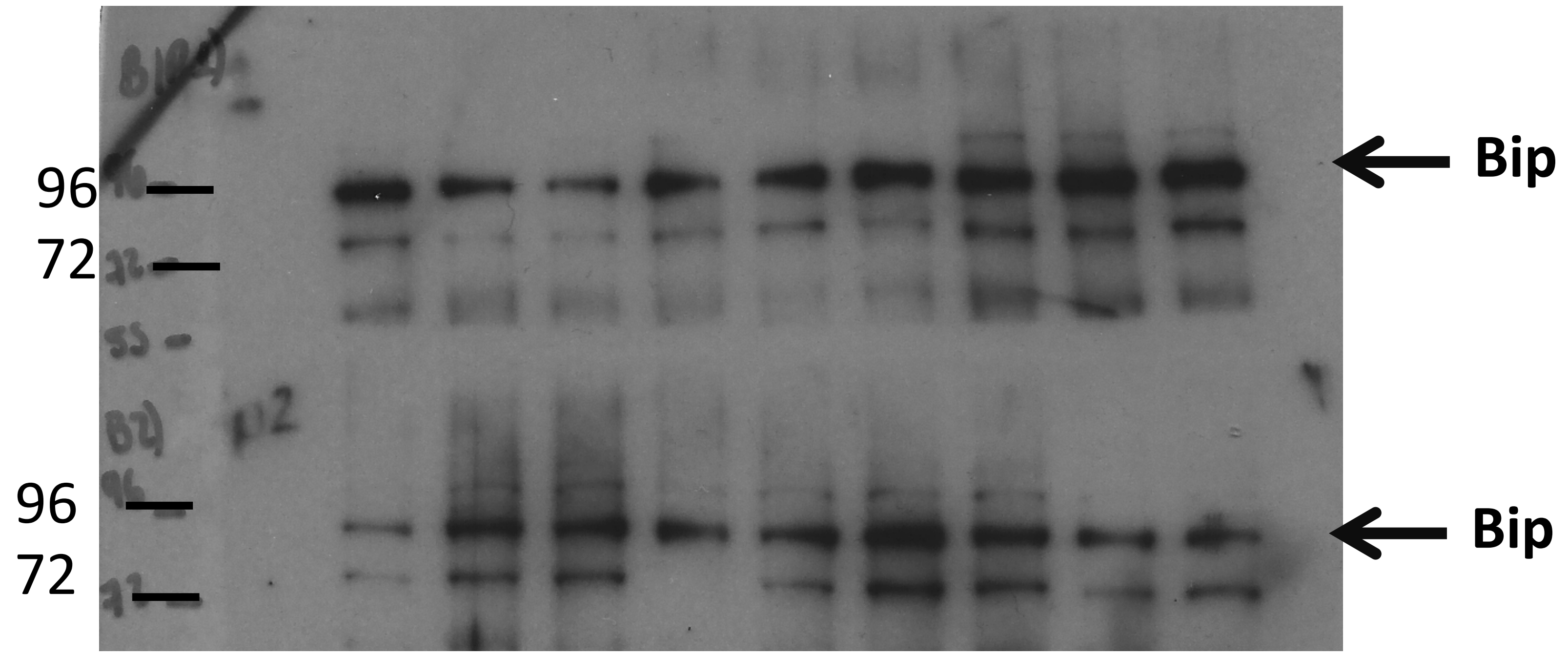

BiP 20ug
1:1,000
1:5,000 ms

β–Actin
MW=42KDa

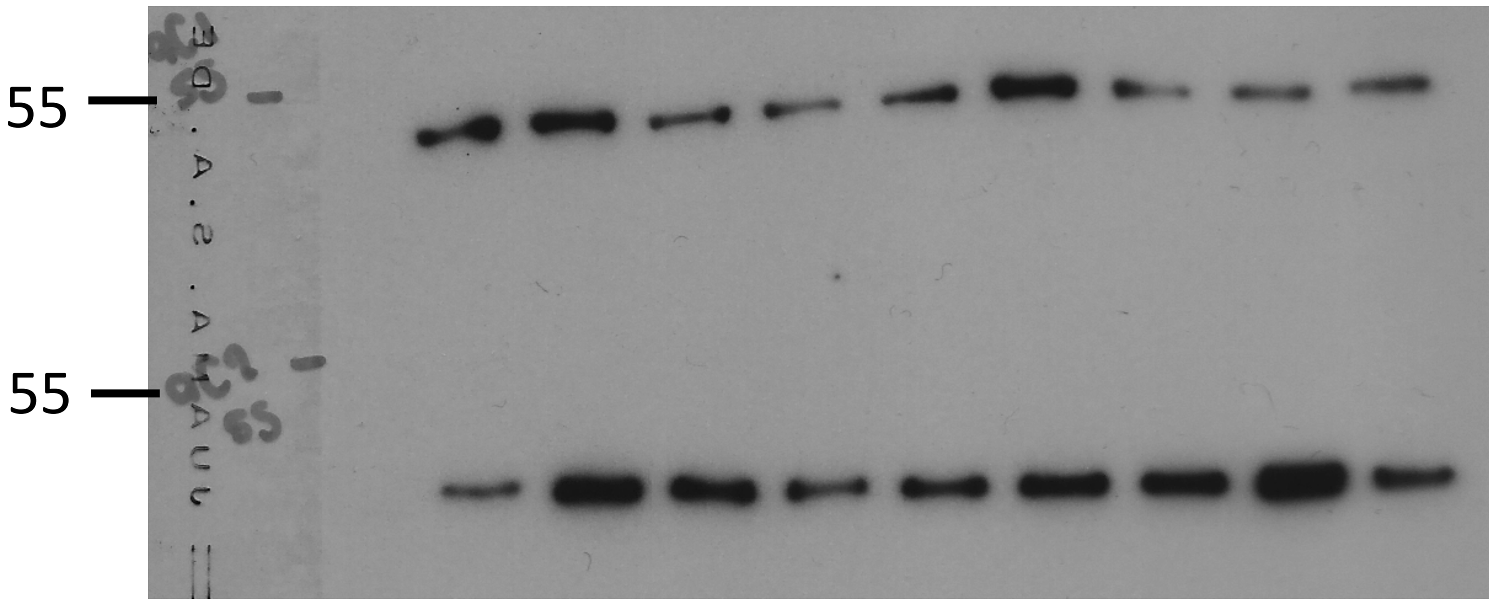

β-actin
1:1,000,000

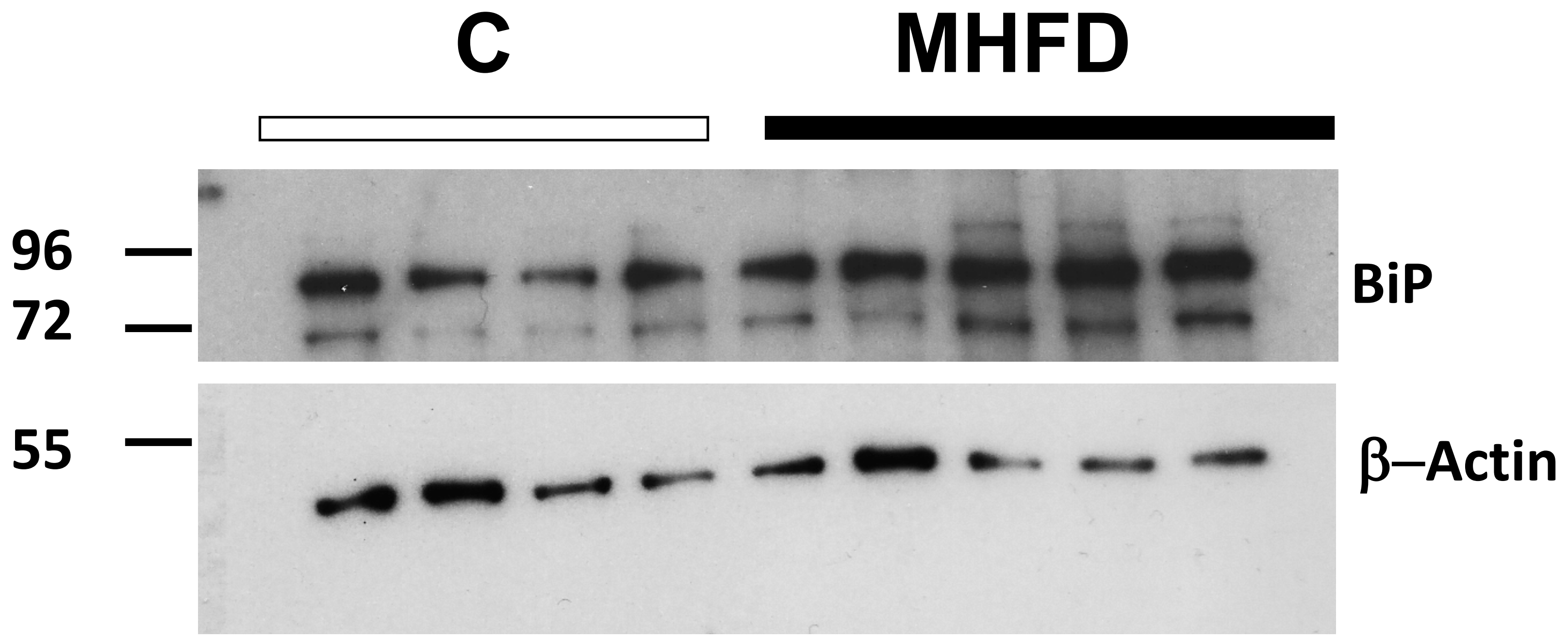

Paper Image

CHOP
MW=27KDa

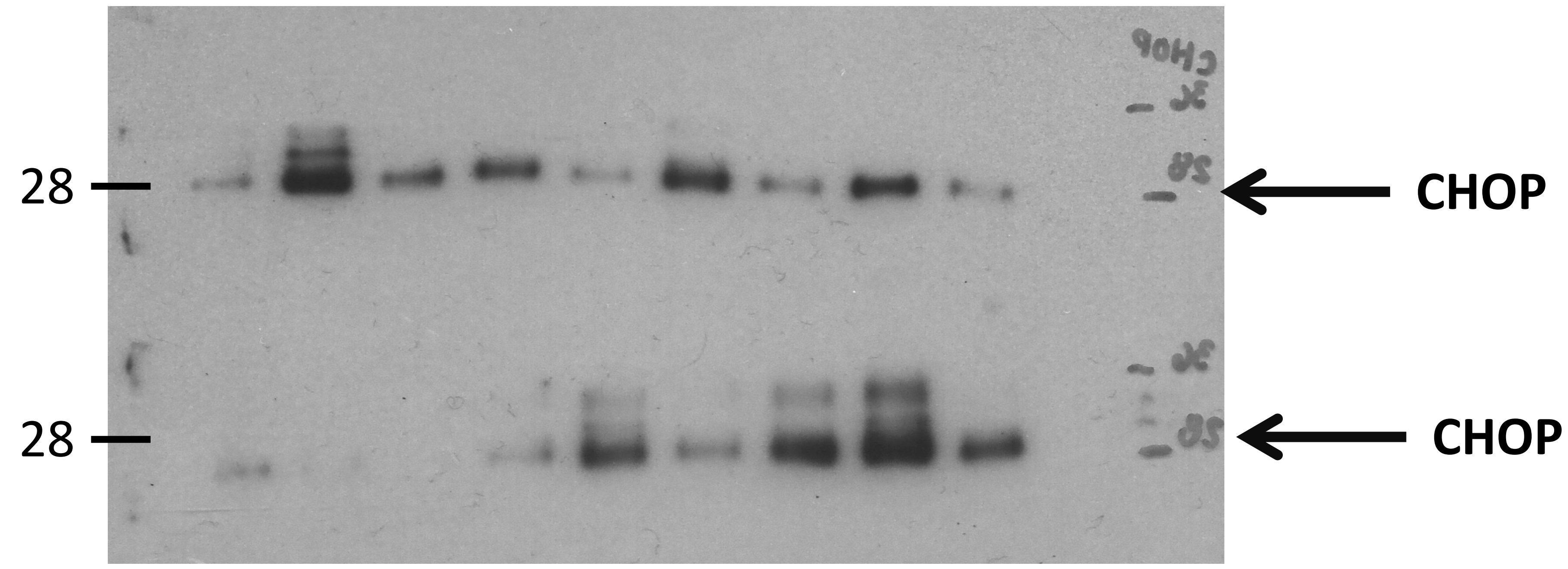

CHOP 20ug
1:5,000
1:5,000 ms

***β*-Actin**
MW=42KDa

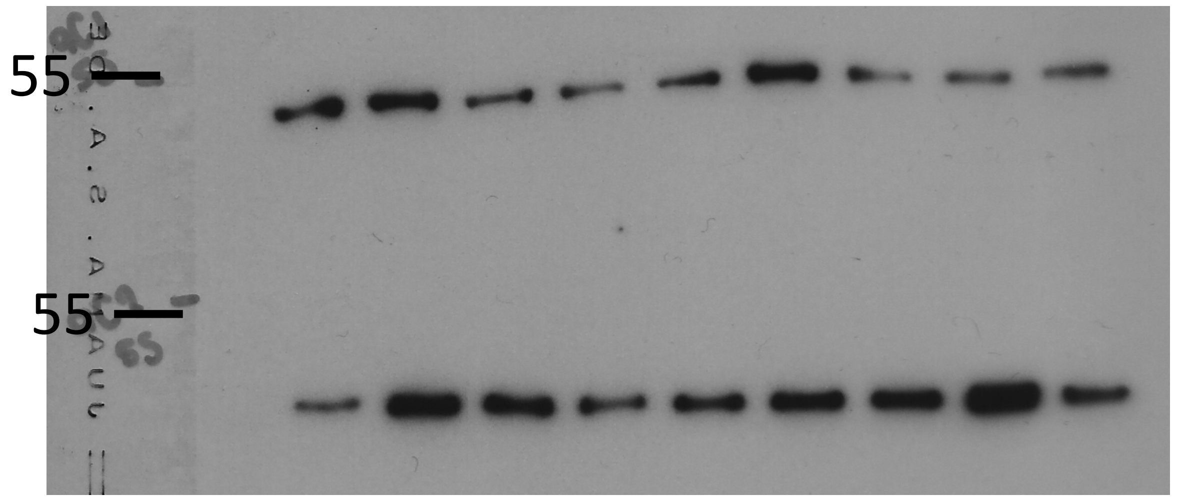

β-actin
1:1,000,000

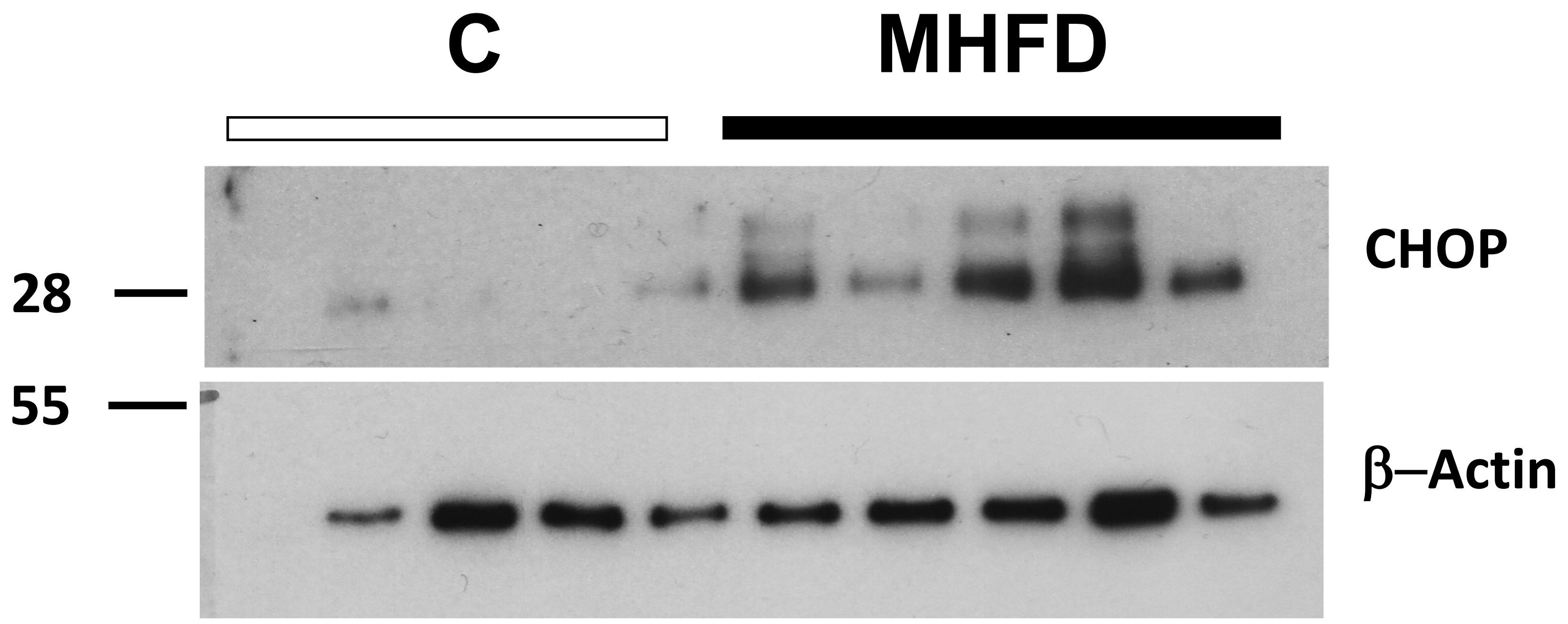

Paper figure

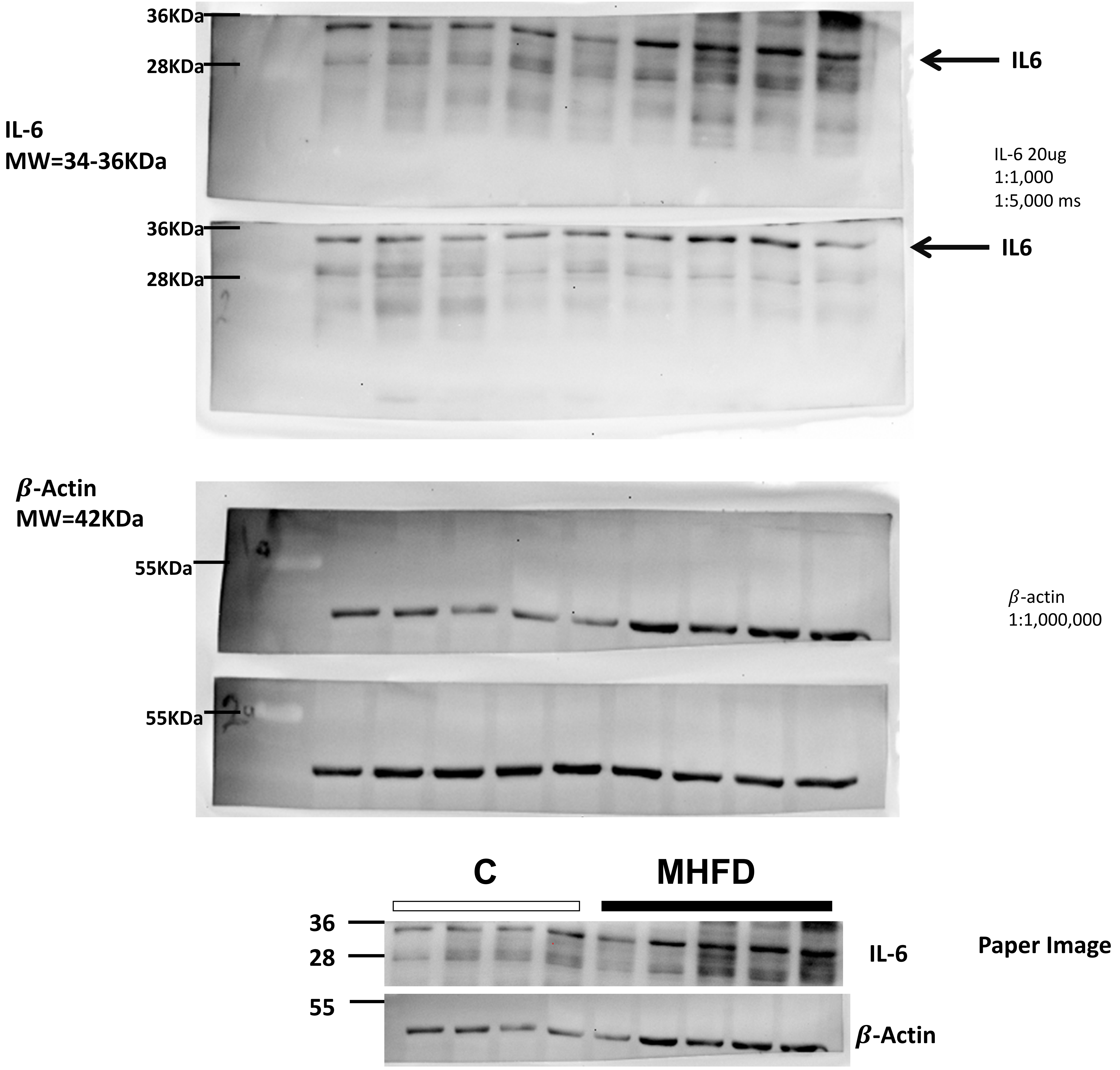
36KDa
28KDa
IL6
IL-6
MW=34-36KDa
IL-6 20ug
1:1,000
1:5,000 ms
36KDa
28KDa
IL6
β-Actin
MW=42KDa
55KDa
β-actin
1:1,000,000
55KDa
C
MHFD
36
28
IL-6
Paper Image
55
β-Actin

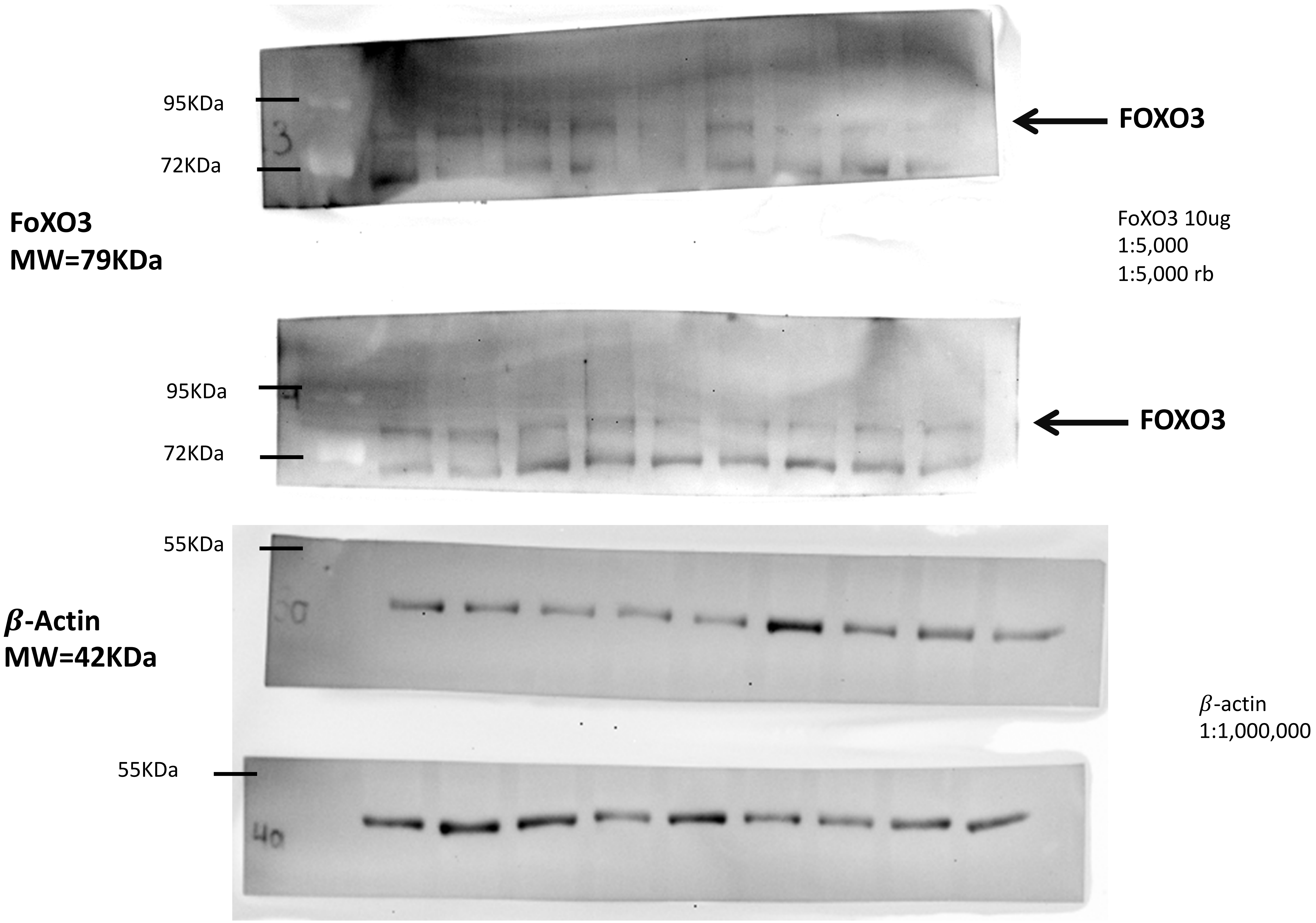
95KDa
72KDa
FOXO3
FoXO3
MW=79KDa
FoXO3 10ug
1:5,000
1:5,000 rb
95KDa
72KDa
FOXO3
55KDa
β-Actin
MW=42KDa
β-actin
1:1,000,000
55KDa

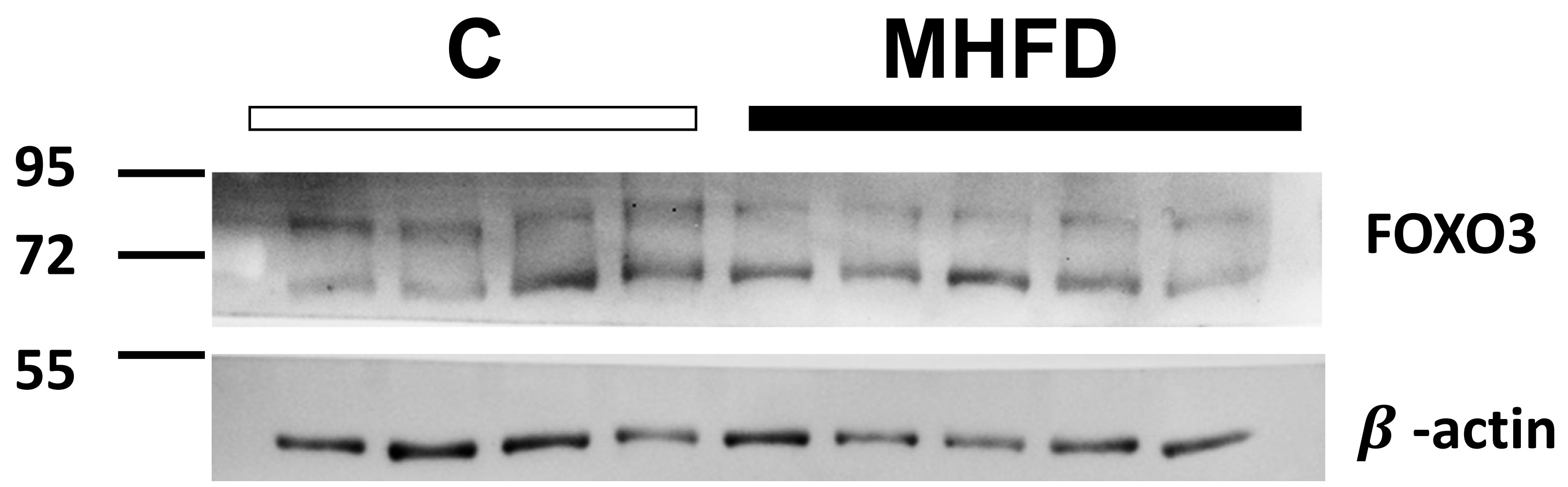
C
MHFD
95
72
FOXO3
55
β -actin

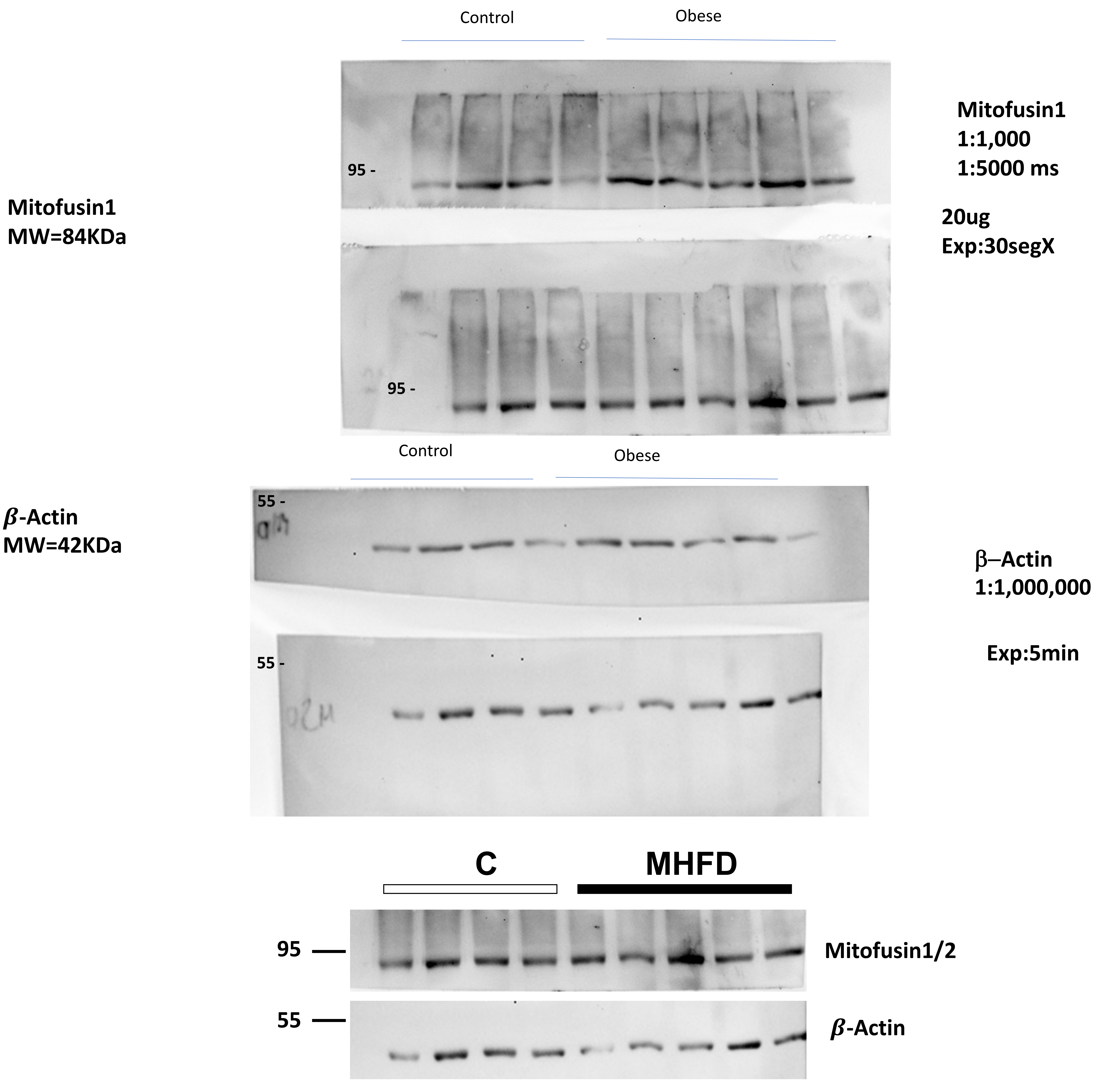
Control
Obese
Mitofusin1
1:1,000
1:5000 ms
20ug
Exp:30segX
Mitofusin1
MW=84KDa
95 -
95 -
Control
Obese
β-Actin
MW=42KDa
55 -
55 -
β–Actin
1:1,000,000
Exp:5min
C
MHFD
95
Mitofusin1/2
55
β-Actin

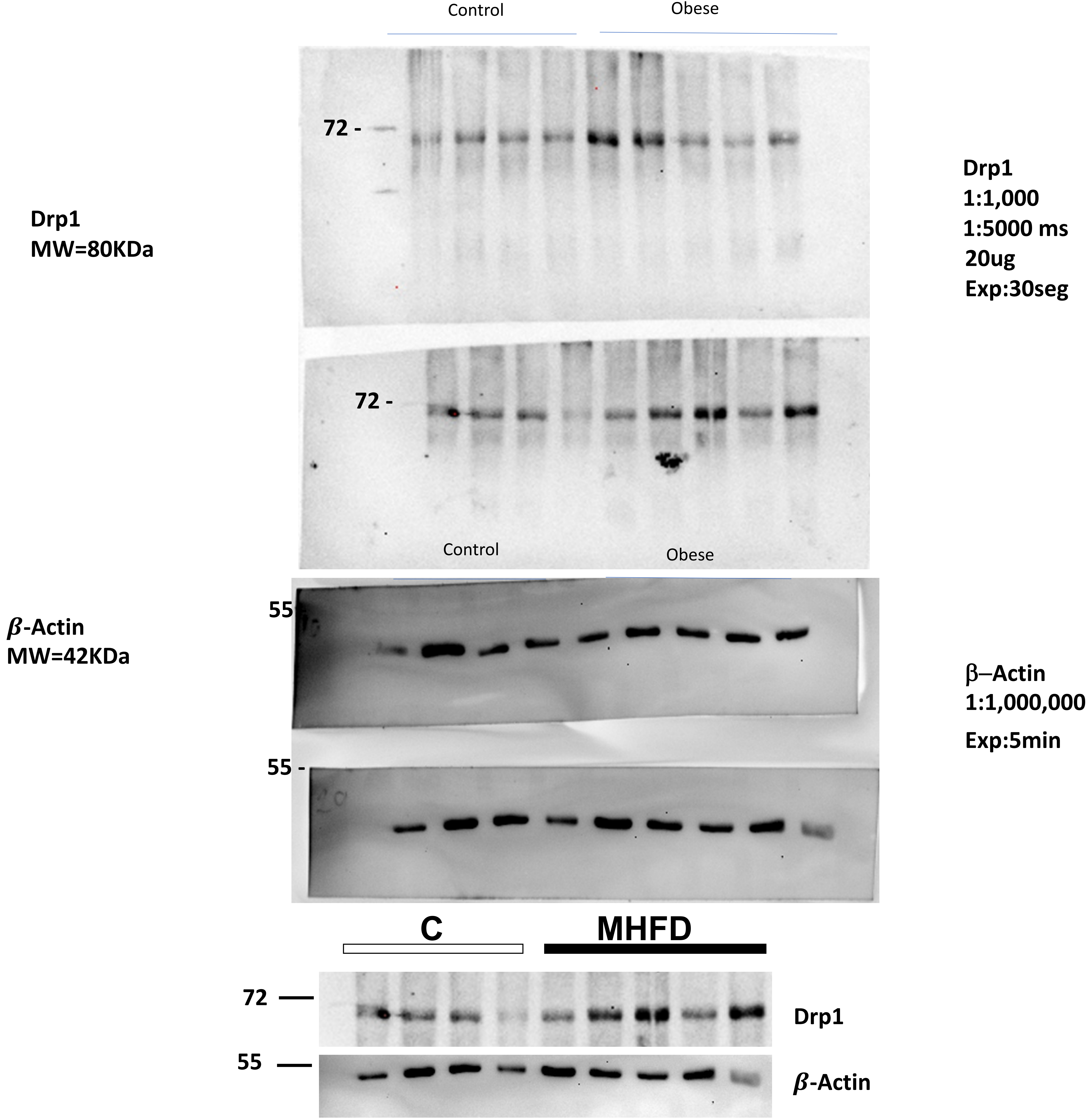

Control
Obese
72 -
Drp1
MW=80KDa
Drp1
1:1,000
1:5000 ms
20ug
Exp:30seg
72 -
Control
Obese
55
β-Actin
MW=42KDa
β–Actin
1:1,000,000
Exp:5min
55 -
C
MHFD
72
Drp1
55
β-Actin
