# Supplementary material for: Early triggers of moderately high‐fat diet‐induced kidney damage
Source: Physiol Rep. 2021 Jul 22;9(14):e14937. doi: 10.14814/phy2.14937 (PMC8295594; doi:10.14814/phy2.14937)
Supplement: Supplementary file 2 — Table S1 [file PHY2-9-e14937-s002.docx]

**Supplementary Table 1.** Physiological parameters in mice fed a MHFD compared to the C group at the end of the study.

| **Parameters** | **C** | **MHFD** | **P** |
| --- | --- | --- | --- |
| Glucose (mg/dL) | 158.5 ± 13.6 | 215.2 ± 12.0 | 0.01 |
| Triglycerides (mg/dL) | 26.1 ± 4.2 | 25.3 ± 2.9 | 0.87 |
| Cholesterol (mg/dL) | 52.3 ± 2.3 | 75.9 ± 3.6 | =<0.001 |
| Fat % | 8.4 ± 0.9 | 33.6 ± 1.5 | =<0.001 |
| Lean % | 91.2 ± 0.9 | 66.2 ± 1.5 | =<0.001 |
| Adipose tissue (g) | 0.4 ± 0.0 | 2.9 ± 0.2 | =<0.001 |

These metabolic results were partially published (11). The group fed a standard diet (C) and the MHFD group. n=10/11 per group, respectively. Data are expressed as means ± SEM, data are statistically different, with p < 0.05.
